# Ethanol Extract of *Ampelopsis brevipedunculata* Rhizomes Suppresses IgE-Mediated Mast Cell Activation and Anaphylaxis

**DOI:** 10.1155/2024/5083956

**Published:** 2024-04-04

**Authors:** Ji-Yeong Park, Min-Jong Kim, Young-Ae Choi, Seung Woong Lee, Soyoung Lee, Yong Hyun Jang, Sang-Hyun Kim

**Affiliations:** ^1^CMRI, Department of Pharmacology, School of Medicine, Kyungpook National University, Daegu 41944, Republic of Korea; ^2^Functional Biomaterial Research Center, Korea Research Institute of Bioscience and Biotechnology, Jeongeup 56212, Republic of Korea; ^3^Department of Dermatology, School of Medicine, Kyungpook National University, Daegu 41944, Republic of Korea

## Abstract

More than 20% of the world's population suffers from allergic diseases, including allergic asthma, rhinitis, and atopic dermatitis that severely reduce the patient's quality of life. The treatment of allergy has been developed, but there are still unmet needs. *Ampelopsis brevipedunculata* (Maxim.) Trautv. is a traditional medicinal herb with beneficial bioactivities, such as antioxidant, anti-hypertension, anti-viral, anti-mutagenic, and skin and liver (anti-hepatotoxic) protective actions. However, its anti-allergic effect has not been addressed. This study designed to investigate the pharmacological effect of an ethanol extract of *A. brevipedunculata* rhizomes (ABE) on mast cell and anaphylaxis models. For *in vivo* studies, we used ovalbumin-induced active systemic anaphylaxis (ASA) and immunoglobulin (Ig) E-mediated passive cutaneous anaphylaxis (PCA) models. In ASA model, oral administration of ABE (1, 10, and 100 mg/kg) attenuated the anaphylactic responses, such as hypothermia, serum histamine, and IgE productions. In PCA model, ABE also suppressed the plasma extravasation and swelling. The underlying mechanisms of action were identified in various mast cell types. *In vitro*, ABE (10, 30, and 60 *µ*g/mL) inhibited the release of essential allergic mediators, such as histamine and *β*-hexosaminidase, in a concentration-dependent manner. ABE prevented the rapid increase in intracellular calcium levels induced by the DNP-HSA challenge. In addition, ABE downregulated the tumor necrosis factor-*α* and interleukin-4 by suppressing the activation of nuclear factor-*κ*B. Collectively, this study is the first to identify the anti-allergic effect of ABE, suggesting that ABE is a promising candidate for treating allergic diseases.

## 1. Introduction

The incidence of allergic diseases, such as allergic rhinitis, asthma, atopic dermatitis, and food allergies, has increased considerably over the past decades, affecting approximately 20% of the world's population [1]. Therefore, addressing allergic diseases is a critical medical field. Currently, the management of allergy includes treatment with anti-histamines, mast cell stabilizers, corticosteroids, and other anti-allergic drugs. However, it is difficult to improve the clinical outcomes of allergic symptoms, and several adverse symptoms (drowsiness and headaches) have been reported for these drugs [2, 3]. Thus, it is urgent to develop new therapeutic agents for allergic diseases.

Immunoglobulin (Ig) E-mediated type I hypersensitivity is regarded as a defining factor in the initiation of allergic diseases and responses [4]. In an inappropriate response, the immune system accepts normally harmless substances as allergens and produces allergen-specific IgE. In turn, IgE binds to Fc*ε*RI, which is a high-affinity IgE receptor that is constitutively present on the mast cell surface [5, 6]. At the next encounter with allergens, the crosslinking of IgE with Fc*ε*RI activates mast cells and releases the newly synthesized and preformed mediators, including histamines, *β*-hexosaminidase, leukotrienes, proteases, prostaglandins, and inflammatory cytokines [7]. Among them, histamine is regarded as one of the critical mediators of allergic responses that induces vasodilation, vascular permeability increase, and leukocyte recruitment followed by edema and hypothermia.

As plants have been an essential resource for the treatment of various diseases through many centuries, the development of traditional herbal medicines can provide a promising platform for new drugs [8, 9]. The grape species *Ampelopsis brevipedunculata* (Maxim.) Trautv. with the common names of creeper, porcelain berry, amur pepper vine, and wild grape is native to temperate areas of Asia [10]. It is currently distributed across China, Korea, Japan, Russia, and America. In East Asian countries, *A. brevipedunculata* has been used as a hemostatic, anti-inflammatory, and analgesic agent in oriental medicine. Its traditional usage has been recorded in several ancient books, including the Chinese Herbal Manual (Chinese ancient book, 1578, by Shih-Chen Li) and Donguibogam (Korean traditional ancient book, 1613, by Jun Heo) [11–13]. According to the Book of Chinese Herbal Medicine (compiled in 1990 by the Commercial Press in China), *A. brevipedunculata* root addresses the problems of arthritis, vomiting, ulcers, diarrhea, and trauma. Based on this knowledge, the pharmacological activities of *A. brevipedunculata,* including bone loss preventive, anti-hepatotoxic, and antioxidant abilities, have been reported [14–16]. In particular, the anti-inflammatory effect of an ethanol extract of *A. brevipedunculata* rhizome (ABE) was demonstrated on an atopic dermatitis model. The results showed that the oral administration of ABE alleviated skin inflammation and reduced the increased serum IgE and histamine levels [12]. Therefore, we expected that ABE inhibits allergic responses. In the present study, the anti-allergic effect of ABE was assessed using both IgE-mediated systemic and local anaphylaxis models and mast cell activation.

## 2. Materials and Methods

A detailed description of the materials and methods used in the present study is provided in Supplementary materials. (available here).

### 2.1. Reagents

The reagents were obtained from the following suppliers: Dulbecco's Modified Eagle's medium (DMEM) (12800-017), *α*-minimum essential medium (MEM) (11900-016), RPMI-1640 (31800-022), and fetal bovine serum (FBS) (16000-044) (Gibco, Grand Island, NY). Antidinitrophenyl (DNP) IgE (D8406), DNP-human serum albumin (HSA) (A6661), ovalbumin (OVA) (A5503), dexamethasone (Dex) (D4902), 4-nitrophenyl-N-acetyl-*β*-D-glucosaminide (N9376), Histodenz (D2158), and o-phthaldialdehyde (P1378) (Sigma-Aldrich, St. Louis, MO). Alum adjuvant (77161) (Thermo Scientific, Waltham, MA) All other reagents were purchased from Sigma-Aldrich unless otherwise stated.

### 2.2. Animals

In the present study, ICR mice (male, 6 weeks of age, and body weight: 22–24 g) and SD rats (male, 10 weeks of age, and body weight: 250–280 g) were obtained from the Dae-Han Experimental Animal Center located in Daejeon, Republic of Korea. The animals were maintained under the following conditions: temperature, 22°C ± 1°C; humidity, 55% ± 5%; light, 12 h light/dark cycles; and air exchanges, 15 times/h. Feed and water were supplied *ad libitum*. All animal experiments were carried out in accordance with the guide for the Public Health Service Policy on the Humane Care and Use of Laboratory Animals and were approved by the Institutional Animal Care and Use Committee of Kyungpook National University (IRB #2022-0002-9).

### 2.3. Cell Culture

Rat basophilic leukemia mast cells (RBL-2H3), rat peritoneal-derived mast cells (RPMCs), and bone marrow-derived mast cells (BMMCs) were cultured in DMEM, *α*-MEM, and RPMI 1640, respectively, with heat-inactivated 10% FBS and 100 U/mL of antibiotics (Hyclone, Logan, UT). All cells were incubated in the condition of a humidified atmosphere of 5% CO_2_ at 37°C. BMMCs were cultured in complete RPMI 1640 with an identical composition to that described in a previous study [17].

### 2.4. OVA-Induced Active Systemic Anaphylaxis (ASA)

The ASA model was established as described in a previous study [18]. For sensitization, the mice were intraperitoneally injected with an OVA mixture consisting of OVA and alum adjuvant on days 0 and 7. Equal amounts of phosphate buffered saline (PBS) were injected into the control group. ABE (1, 10, and 100 mg/kg) and Dex (10 mg/kg) were orally administrated on days 9, 11, and 13. Mice received antigens *via* an intraperitoneal injection of OVA (200 *µ*g), and rectal temperature was measured at 10 min intervals for 80 min on day 14. The mice were sacrificed, and blood was collected.

### 2.5. IgE-Mediated Passive Systemic Anaphylaxis (PCA)

The PCA model was induced using the methods described in a previous study [18]. First, 0.5 *μ*g of anti-DNP IgE was intradermally injected into each site of the mouse ears for sensitization. Two days later, ABE (1, 10, and 100 mg/kg) and Dex (10 mg/kg) were orally administrated 1 h before the antigen injection. Mice were challenged by the intravenous injection of a mixture of 100 *µ*L of DNP-HSA (10 mg/mL) and 100 *µ*L of 4% Evans blue. After 30 min, the ears were excised and incubated with 1 mL of 1 M KOH for 3 days, 4 mL of a mixture with acetone and phosphoric acid (5 : 13) was added, and the supernatant was centrifuged. The absorbance was measured at 620 nm using a spectrophotometer (Molecular Devices).

### 2.6. RPMC Preparation

Peritoneal mast cells were obtained as described previously [19]. Briefly, 50 mL of Tyrode's buffer was infused into the abdomen. After massage, the buffer was collected using a Pasteur pipette. The supernatant was resuspended in 1 mL of Tyrode's buffer and centrifuged. For mast cell purification, the suspension was layered on top of the Histodenz solution, spun down, and washed. The isolated cells exhibited a purity of approximately 95% for mast cells in toluidine blue staining and more than 97% viability in trypan blue staining.

### 2.7. BMMC Preparation

BMMCs were established as described previously [20]. Mouse femurs and tibia were separated from muscles. The internal capacity of the bone was exposed, and the bone marrow was flushed with 1 mL of culture media, transferred to a T-flask 4 days later, and cultured. Further experiments were conducted after confirming that the cells expressed >90% of Fc*ε*RI^+^ and c-kit^+^.

### 2.8. Histamine and *β*-Hexosaminidase Assay

For sensitization, RBL-2H3 (5 × 10^5^ cells/well in a 12-well plate), RPMCs (2 × 10^4^ cells/well in a 24-well plate), and BMMCs (5 × 10^5^ cells/well in a 12-well plate) were incubated overnight with IgE (50 ng/mL) and treated with ABE (10, 30, and 60 *µ*g/mL) or Dex (10 *µ*M). After 1 h, the cells were treated with DNP-HSA (100 ng/mL) for 4 h (RBL-2H3) or 30 min (RPMCs and BMMCs). The culture media were collected, and attached cells were dissolved in 0.5% Triton X-100. Histamine and *β*-hexosaminidase levels were determined according to a previously described method [21]. To quantify the histamine contained in the serum and culture media, 0.1 N HCl and 60% perchloric acid were mixed with the samples, which were then centrifuged. The supernatant was transferred to fresh a tube, and 5 M NaCl, 5 N NaOH, and *n*-butanol were added before the second centrifugation. Next, 0.1 N HCl and *n*-heptane were mixed with the supernatant. Histamine was quantified using *o*-phthaldialdehyde spectrofluorometry. *β*-Hexosaminidase release was measured according to the procedure described in a previous study [22]. Briefly, 40 *µ*L of media and cell lysate was incubated at 37°C for 1 h with substrate buffer, and the absorbance was measured at 405 nm using a spectrophotometer (Molecular Devices).

### 2.9. Intracellular Calcium Level Determination

Intracellular calcium levels were determined using Fluo-3/AM (Invitrogen, Carlsbad, CA) as described in a previous study [17]. RBL-2H3 (2 × 10^4^ cells/well in a 12-well plate) were incubated overnight with IgE (50 ng/mL) and then incubated with Fluo-3/AM (5 *µ*M) for 1 h at 37°C. Subsequently, the cells were washed and pretreated with ABE (10, 30, or 60 *µ*g/mL) or BAPTA-AM (10 *µ*M) before the DNP-HSA (100 ng/mL) challenge. Fluorescence intensity was detected using a fluorescent plate reader.

### 2.10. qPCR

Anti-DNP IgE (50 ng/mL)-sensitized RBL-2H3 (5 × 10^5^ cells/well in a 12-well plate) were pretreated with ABE (10, 30, or 60 *µ*g/mL) or Dex (10 *µ*M) and stimulated with DNP-HSA (100 ng/mL) for 1 h. Total cellular RNA was purified using a RNAiso Plus kit (Takara Bio, Shiga, Japan), and complementary DNA (cDNA) was synthesized from 1 *µ*g of RNA using a Maxime RT PreMix Kit (iNtRON Biotech, Sungnam, Republic of Korea). qPCR was conducted using a Thermal Cycler Dice TP850 (Takara Bio). TP850 software was used for relative quantification of the target genes. The primer sequences are shown in Supplementary Table S1.

### 2.11. Western Blot

Activation of NF-*κ*B was assessed by Western blot. Anti-DNP IgE (50 ng/mL)-sensitized RBL-2H3 (1.5 × 10^6^ cells/well in a 6-well plate) were pretreated with ABE (60 *µ*g/mL) or Dex (10 *µ*M) and stimulated with DNP-HSA (100 ng/mL) for 1 h. Nuclear and cytosolic protein extracts were obtained as previously described [22]. Equal amounts of protein were separated by electrophoresis on a 7.5–10% sodium dodecyl sulfate-polyacrylamide gel. After being transferred, the nitrocellulose membrane was incubated overnight with specific primary antibodies and then detected with horseradish peroxidase-conjugated secondary antibodies. Detailed information of the antibodies used in the assay is described in Supplementary Table S2. Chemiluminescent substrate (Thermo Scientific) was used for target protein detection.

### 2.12. Statistical Analysis

Statistical analyses were carried out in GraphPad Prism 5 (GraphPad Software Inc., San Diego, CA) using one-way analysis of variance followed by Tukey's post-test. Significance was set at *P* < 0.05. Data are presented as the mean ± standard error of the mean (SEM).

## 3. Results

### 3.1. Effects of ABE on Anaphylaxis Models

**Figure 1:**
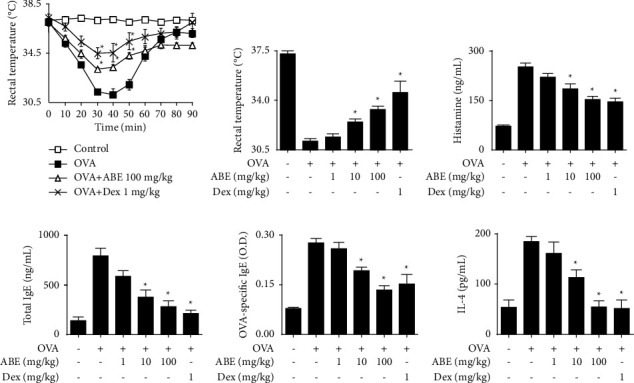
Effects of ABE on the OVA-induced active systemic anaphylaxis model. (a) The rectal temperature of mice was measured for 80 min after the OVA challenge. (b) Bar graph showing the rectal temperature of the mice at 40 min after the OVA challenge. (c–f) The serum histamine, total IgE, OVA-specific IgE, and IL-4 levels were determined using ELISA. The graph data represent the mean ± SEM (*n* = 5/group). ^*∗*^*p* < 0.05 compared with the OVA-challenged group. Dex: dexamethasone.

**Figure 2:**
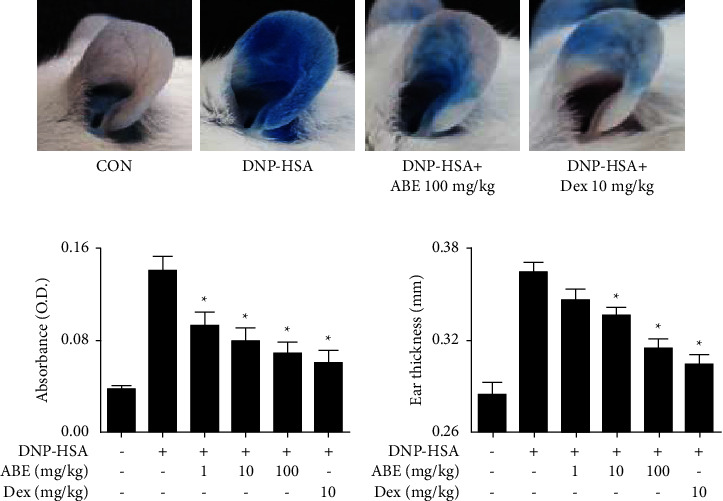
Effects of ABE on the IgE-mediated passive cutaneous anaphylaxis model. (a) Representative photographic images show Evans blue pigmentation. (b) The optical density of lysed ear tissues was measured to determine the amount of dye. (c) The graph represents the average of ear thickness. The graph data represent the mean ± SEM (*n* = 5/group). ^*∗*^*p* < 0.05 compared with the anti-DNP IgE-sensitized group. Dex: dexamethasone.

Anaphylaxis manifests as type I hypersensitivity and is one of the most serious allergic reactions [18]. To evaluate the anti-allergic effects of ABE *in vivo*, systemic and local anaphylaxis models were used. ASA was induced by OVA injection into mice that had been sensitized with alum adjuvant and OVA. As a result, a decrease in rectal temperature was observed from 20 to 60 min after the OVA challenge, which was prevented by oral administration of ABE (Figure 1(a)). Figure 1(b) shows that ABE significantly suppressed the OVA-induced hypothermia 40 min after OVA injection. In addition, ABE reduced the increased serum histamine, total IgE, OVA-specific IgE, and IL-4 levels in a dose-dependent manner (Figures 1(c)–1(f)). PCA reactions were induced by the intravenous injection of DNP-HSA into anti-DNP IgE-sensitized mice. As a result, Figure 2(a) visually shows that oral administration of ABE suppressed the Evans blue extravasation. The data were confirmed by measuring the absorbance of lysed ear tissues (Figure 2(b)). ABE also suppressed the increased ear thickness (Figure 2(c)).

### 3.2. Effects of ABE on Degranulation and Calcium Influx in Mast Cells

**Figure 3:**
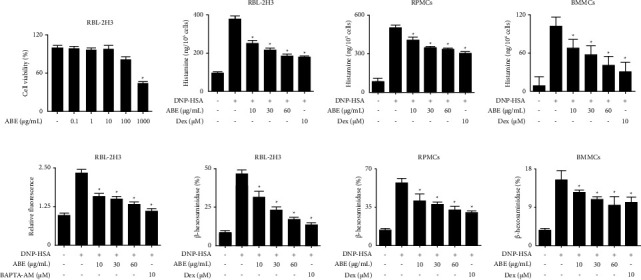
Effects of ABE on degranulation and intracellular calcium influx in mast cells. (a) RBL-2H3 were incubated with ABE for 12 h. Cell viability was determined using MTT assay and revealed as the relative absorbance of control cells and ABE treated cells. (b–d, f–h) Anti-DNP IgE-sensitized mast cells were pretreated with/without drugs (ABE or Dex) for 1 h. The cells were stimulated with DNP-HSA for 4 h (RBL-2H3) or 30 min (BMMCs and RPMCs). The released histamine was purified and detected using a fluorescent plate reader. The percentage of *β*-hexosaminidase release was calculated. (e) Anti-DNP IgE-sensitized RBL-2H3 were incubated with Fluo-3/AM for 1 h, treated with/without ABE or BAPTA-AM for 1 h, and challenged with DNP-HSA. Graph data represent the relative intracellular calcium levels compared with those of control cells. The graph data represent the mean ± SEM. ^*∗*^*p* < 0.05 compared with the DNP-HSA-stimulated group. Dex: dexamethasone.

The primary cultured mast cells are attractive means of mast cell research relevant to human allergic diseases because they can express the physiological properties of mast cell *in vivo*. Therefore, the *in vitro* experiment used three types of mast cells (RBL-2H3, RPMCs, and BMMCs). Prior to investigating the effects of ABE, cytotoxicity was tested by incubating RBL-2H3 with various concentration of ABE (0.1–1,000 *μ*g/mL) for 12 h. No cytotoxicity was observed up to 100 *μ*g/mL (Figure 3(a)). Therefore, ABE was used at concentrations that excluded cytotoxicity. To evaluate the inhibitory effect of ABE on mast cell degranulation, we evaluated the release of histamine and *β*-hexosaminidase. In this study, mast cells were sensitized by overnight incubation with anti-DNP IgE and stimulated with DNP-HSA. ABE significantly inhibited the high levels of histamine and *β*-hexosaminidase in RBL-2H3, RPMCs, and BMMCs (Figures 3(b)–3(d) and 3(f)–3(h)). Mast cell degranulation depends on intracellular calcium, which acts as a secondary messenger [23]. To examine the mechanism of action, Fluo-3/AM was used to detect the intracellular calcium levels. In our study, the increased calcium levels were significantly reduced by ABE (Figure 3(e)).

### 3.3. Effects of ABE on Inflammatory Cytokines and NF-*κ*B in Mast Cells

**Figure 4:**
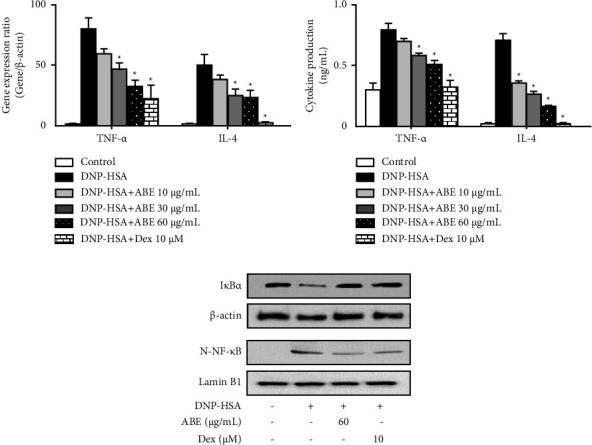
Effects of ABE on inflammatory cytokine expression and NF-*κ*B activation in mast cells. Anti-DNP IgE-sensitized RBL-2H3 were pretreated with/without drugs (ABE or Dex) for 1 h and stimulated with DNP-HSA. (a) After 1 h, the expression of TNF-*α* and IL-4 was determined using qPCR. The graph data represent the relative target gene expression. (b) After 6 h, inflammatory cytokine secretion was measured using ELISA. The graph data represent the mean ± SEM. (c) Western blot images show bands for I*κ*B*α* and NF-*κ*B obtained from cytoplasmic and nuclear protein extraction, respectively. *β*-Actin and Lamin B1 were used as loading controls. ^*∗*^*p* < 0.05 compared with the DNP-HSA-stimulated group. Dex: dexamethasone.

In the late phase of allergic reactions, IgE-sensitized mast cells propagate the inflammatory response by continuously producing and releasing proinflammatory cytokines [24]. Our results showed that ABE markedly inhibited the gene expression and cytokine secretion of TNF-*α* and IL-4 in mast cells (Figures 4(a) and 4(b)). NF-*κ*B has long been regarded as an essential transcription factor that regulates the expression of inflammatory genes. Therefore, NF-*κ*B activation was assayed using Western blot to clarify the mechanisms of action of ABE. I*κ*B*α* degradation and nuclear translocation of NF-*κ*B were observed in activated mast cells, which were reduced by ABE treatment (Figure 4(c)).

## 4. Discussion

As mentioned earlier, IgE-mediated type I hypersensitivity causes anaphylaxis and manifests as a variety of allergic diseases [25]. Mast cells are primary effector cells that exert critical roles in allergic reactions by secreting diverse biological mediators [26, 27]. Therefore, the suppression of IgE-mediated anaphylaxis and mast cell activation can be a central strategy for the development of new therapeutic agents for allergic diseases. *A. brevipedunculata* has a long history as medicinal herb in oriental medicine, and its various pharmacological actions have recently been revealed. However, the anti-allergic effect of *A. brevipedunculata* has not yet been addressed. Collectively, we tested the inhibitory effect of ABE on an anaphylaxis model and on IgE-mediated mast cell activation.

The ASA and PCA models are suitable anaphylaxis models for the identification of anti-allergic agents or the study of the mechanisms of allergic reactions. Both models show allergic symptoms under the influence of activated mast cells and basophils [28]. In ASA, OVA sensitization enhances IgE production in the serum; subsequently, OVA challenge triggers anaphylactic reactions related to IgE-sensitized mast cell activation in the mouse body. In this regard, this model is accompanied by an elevation in serum histamine following vasodilation and hypothermia, which are related to anaphylaxis severity [29]. In PCA, the DNP-HSA challenge eventually causes mast cell activation and results in vascular hyperpermeability and plasma extravasation, which are associated with urticaria features [27]. In our study, the oral administration of ABE suppressed the IgE-mediated anaphylactic responses in both the ASA and PCA models. These results suggest that ABE suppresses the mast cell activation *in vivo* and thus reduces the systemic and local anaphylactic response. Moreover, the inhibitory effects of ABE were confirmed in *in vitro* studies.

Histamine is a biogenic amine that is recognized as an essential factor in allergic responses [30, 31]. *β*-Hexosaminidase is predominantly present in mast cell granules and also causes the allergic inflammatory response [32]. In fact, histamine and *β*-hexosaminidase are the first molecules released from granules and are engaged in the rapid action of mast cells [33]. Therefore, we evaluated the histamine and *β*-hexosaminidase levels in three types of mast cells. Our results revealed that ABE significantly inhibited IgE-mediated mast cell degranulation. In addition, previous studies reported that mast cell degranulation is regulated by intracellular Ca^2+^ dynamics. The interaction of allergens with IgE/Fc*ε*RI initiates Ca^2+^ influx from endoplasmic reticulum stores and several channels present in the plasma membrane. Ca^2+^ elevation activates a series of downstream events that lead to the secretion of a great number of granules [34, 35]. In our study, ABE reduced the sharply increased intracellular calcium levels in activated mast cells. Therefore, it is predicted that ABE inhibits IgE-mediated mast cell degranulation by modulating intracellular Ca^2+^ influx.

Mast cells also release inflammatory cytokines that promote the progression of allergic inflammatory responses by recruiting circulating leukocytes [36]. Mast cell-derived TNF-*α* is a potent multifunctional cytokine and is emerging as a key player in allergic inflammation [37]. IL-4 participates in the differentiation of T-cells into Th2 cells. In addition, it is involved in the activation, proliferation, and differentiation of B lymphocytes, thereby promoting IgG_1_ and IgE production [38]. Therefore, regulation of TNF-*α* and IL-4 expression is considered an important qualification for the treatment of allergic inflammation [39]. Our data showed that ABE significantly reduced the expression of TNF-*α* and IL-4 in activated mast cells, suggesting that ABE is anticipated to inhibit mast cell-mediated allergic inflammation. In the normal state, NF-*κ*B dimers interact with I*κ*B proteins and persist in an inactive form. When an antigen binds to the IgE/Fc*ε*RI complex, I*κ*B is degraded upon its phosphorylation by I*κ*B kinase (IKK), thus allowing the translocation of NF-*κ*B into the nucleus where it acts as a transcription factor of target genes encoding inflammatory cytokines, chemokines, and adhesion molecules [40]. In this study, ABE suppressed I*κ*B degradation and NF-*κ*B translocation in activated mast cells. Collectively, these results indicated that ABE inhibits the expression of inflammatory cytokines through the suppression of NF-*κ*B activation. The *in vitro* results indicated that ABE inhibits IgE-mediated mast cell degranulation and activation, thus predicting that ABE may alleviate mast cell-mediated allergic inflammation.

In our previous study, the HPLC profile of ABE implied that it contains various beneficial compounds, including catechin, gallic acid, and resveratrol [12]. Catechins are well-known polyphenolic flavanols with a variety of positive effects on the human body, such as anti-inflammatory, antioxidant, anti-cancer, and anti-diabetic properties [41]. In addition, a previous study demonstrated that catechin inhibited the PCA reaction (type I allergic reaction) and histamine release from rat peritoneal exudate cells [42, 43]. Moreover, the inhibitory effect of gallic acid and resveratrol on histamine and proinflammatory cytokine release in activated mast cells has been demonstrated [44, 45].We compared the effect of ABE with catechin, gallic acid, and resveratrol. Gallic acid showed the greatest inhibitory effect (Supplementary Figure S1).

## 5. Conclusion

Our data showed that the oral administration of ABE reduced the systemic and local anaphylactic reactions in mouse models. The underlying mechanism of action was demonstrated using an *in vitro* study. Thus, we propose that ABE can be a promising therapeutic candidate for various allergic diseases. However, more detailed research is needed to use ABE as a pharmacotherapy in clinical practice.

## Data Availability

The data used and analyzed in this study are included within the article and the supplementary information files.

## Supplementary Materials

Supplementary Materials Supplementary material consists of the additional methods and the detailed information of primer sequences and antibodies used in this study.
