# Supplementary material for: Ethanol Extract of Ampelopsis brevipedunculata Rhizomes Suppresses IgE-Mediated Mast Cell Activation and Anaphylaxis
Source: Adv Pharmacol Pharm Sci. 2024 Apr 4;2024:5083956. doi: 10.1155/2024/5083956 (PMC11008974; doi:10.1155/2024/5083956)
Supplement: Supplementary Materials — Supplementary material consists of the additional methods and the detailed information of primer sequences and antibodies used in this study. [file 5083956.f1.docx]

**Supplementary material**

**Materials and Methods**

*ABE preparation and identification*

*A. brevipedunculata* rhizome were procured from an herbal store in Seoul, Republic of Korea (A voucher specimen KRIBB-KR2013-1). We previously showed that procedure of *A. brevipedunculata* extraction and the structures identification of components using spectroscopic analyses [1]. Briefly, the dried and pulverized materials were extracted with 95% ethanol and evaporated in vacuo. The yield of ABE was approximately 3.7%. To verify the component structure, the extract was suspended in H_2_O, progressively partitioned with ethyl acetate, separated using silica gel column chromatography. Seventeen compounds were yielded using MPLC (C18 and silica gel) and semi-preparative HPLC. The NMR data of isolated compounds and reported literature data confirmed the ABE components.

*Cell viability*

Cell viability was assessed using a MTT assay (WelGENE, Seoul, Republic of Korea) as previously described. RBL-2H3 (6 × 10^4^ cells/well in a 96-well plate) were incubated with ABE (1, 10, 100, and 1,000 µg/mL) for 12 h, and MTT reagent (1 mg/mL) was added. After 2 h, the formed formazan crystals were dissolved by dimethyl sulfoxide. The absorbance was measured at a wavelength of 570 nm using a spectrophotometer (Molecular Devices).

*Histamine and β-hexosaminidase assay*

For sensitization, RBL-2H3 (5 × 10^5^ cells/well in a 12-well plate) were incubated overnight with IgE (50 ng/mL) and treated with ABE (60 µg/mL), catechin, gallic acid, resveratrol, or Dex (10 µM). After 1 h, the cells were treated with DNP-HSA (100 ng/mL) for 4 h. The culture media were collected, and attached cells were dissolved in 0.5% Triton X-100. Histamine levels were determined according to a previously described method [2]. First, 0.1 N HCl and 60% perchloric acid were mixed with the cultured media, which were then centrifuged. The supernatant was transferred to fresh a tube, and 5 M NaCl, 5 N NaOH, and *n*-butanol were added before the second centrifugation. Next, 0.1 N HCl and *n*-heptane were mixed with the supernatant. Histamine was quantified using *o*-phthaldialdehyde spectrofluorometry. β-hexosaminidase release was measured according to the procedure described in a previous study [3]. Briefly, 40 µL of media and cell lysate was incubated at 37ºC for 1 h with substrate buffer, and the absorbance was measured at 405 nm using a spectrophotometer (Molecular Devices).

*ELISA*

Serum IgE and cytokine levels were determined by ELISA. Anti-DNP IgE (50 ng/mL)-sensitized RBL-2H3 (5 × 10^5^ cells/well in a 12-well plate) were pretreated with ABE (10, 30, or 60 µg/mL) or Dex (10 µM) and stimulated with DNP-HSA (100 ng/mL) for 6 h. The concentration of target proteins was evaluated using specific kits (BD Biosciences, San Diego, CA, USA) following the manufacturer’s instructions. In case of OVA-specific IgE, OVA (20 µg/mL) was used instead of capture antibody.

**Supplementary Tables**

**Table S1.** Primers sequences used in qPCR.

| Primer | Sequence 5'→3' | GenBank accession number |
| --- | --- | --- |
| β-actin | F: GAAGCTGTGCTATGTTGCCCTAGA | NM_031144.3 |
|  | R: GTACTCCTGCTTGCTGATCCACAT |  |
| TNF-α | F: TCCCAAATGGGCTCCCTCTC | NM_012675.3 |
|  | R: AAATGGCAAACCGGCTGACG |  |
| IL-4 | F: TGCACCGAGATGTTTGTACCAGA | NM_201270.1 |
|  | R: TTGCGAAGCACCCTGGAAG |  |

F, forward; R, reverse.

**Table S2.** Antibodies used for Western blot.

| Target | Supplier | Cat. No. | Size (kDa) | Host | Dilution |
| --- | --- | --- | --- | --- | --- |
| NF-κB | Santa Cruz | sc-109 | 65 | Rabbit | 1:1000 |
| IκBα | Santa Cruz | sc-371 | 27 | Rabbit | 1:1000 |
| β-actin | Santa Cruz | sc-8432 | 42 | Mouse | 1:1000 |
| Lamin B1 | Santa Cruz | sc-6217 | 65 | Goat | 1:1000 |
| Rabbit IgG | Cell Signaling | 7074S |  | Goat | 1:2000 |
| Mouse IgG | Cell Signaling | 7076S |  | Horse | 1:2000 |
| Goat IgG | Santa Cruz | sc-2354 |  | Mouse | 1:2000 |

**
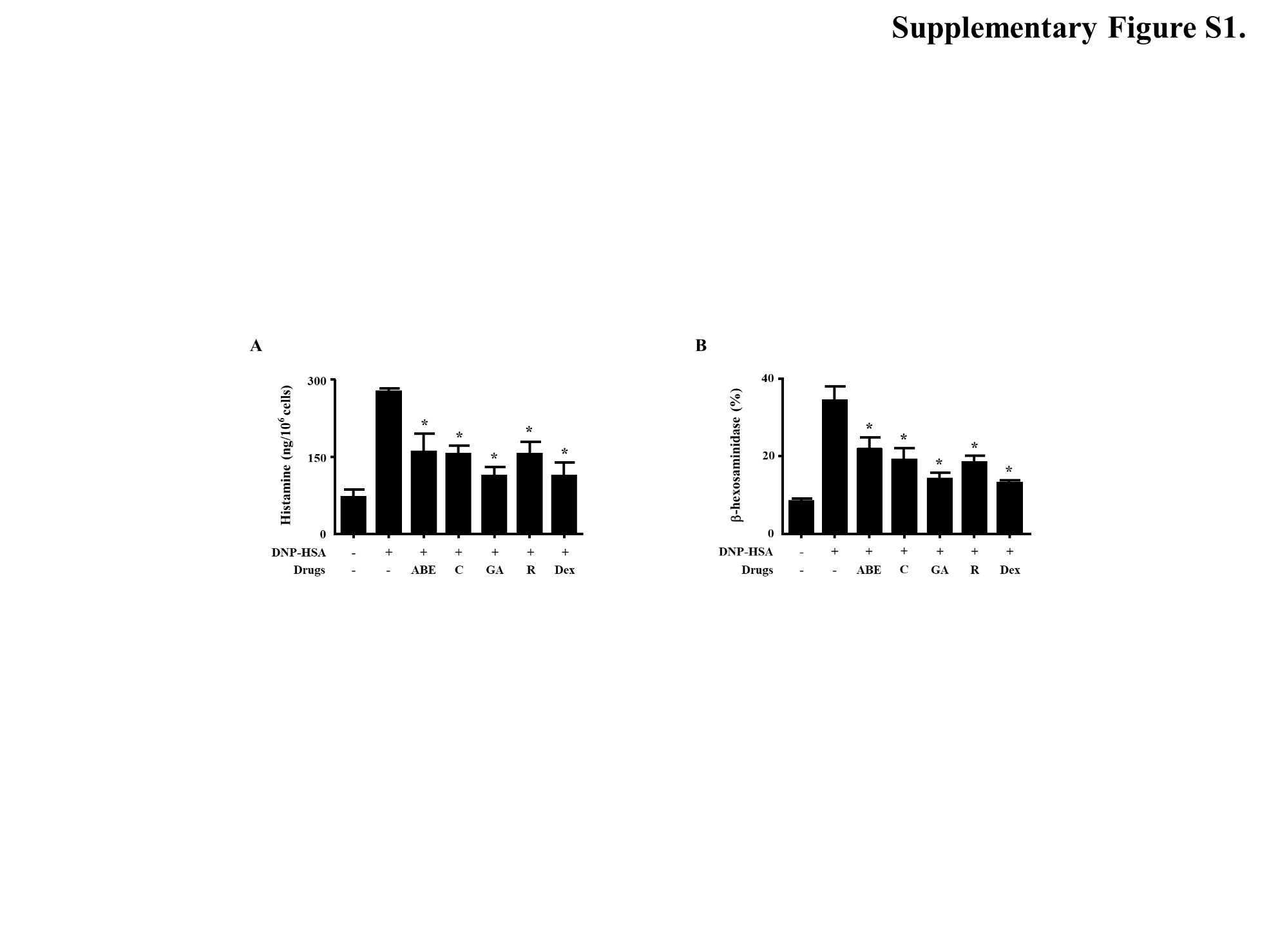
Supplementary Figure**

**FIGURE S1.** Comparison of the effects of ABE and its components in mast cells.

Anti-DNP IgE-sensitized mast cells were pretreated with/without ABE (60 μg/mL) or its components (C, GA, R, or Dex; 10 μM) for 1 h. RBL-2H3 were stimulated with DNP-HSA for 4 h. The released histamine was purified and detected using a fluorescent plate reader. The percentage of β-hexosaminidase release was calculated. The graph data represent the mean ± SEM. **p* < 0.05 compared with the DNP-HSA-stimulated group. ABE: Ethanol extract of *A. brevipedunculata* rhizomes, C: catechin, GA: gallic acid, R: resveratrol, Dex: dexamethasone.

**Reference**

1. Choi, Y.A., J.H. Yu, H.D. Jung, S. Lee, P.H. Park, H.S. Lee, T.K. Kwon, T.Y. Shin, S.W. Lee, M.C. Rho, Y.H. Jang, and S.H. Kim, Inhibitory effect of ethanol extract of *Ampelopsis brevipedunculata* rhizomes on atopic dermatitis-like skin inflammation. J. Ethnopharmacol., 2019. **238**: p. 111850.

2. Dhakal, H., E.J. Yang, S. Lee, M.J. Kim, M.C. Baek, B. Lee, P.H. Park, T.K. Kwon, D. Khang, K.S. Song, and S.H. Kim, Avenanthramide C from germinated oats exhibits anti-allergic inflammatory effects in mast cells. Sci. Rep., 2019. **9**(1): p. 6884.

3. Kim, M.J., I.G. Je, J. Song, X. Fei, S. Lee, H. Yang, W. Kang, Y.H. Jang, S.Y. Seo, and S.H. Kim, SG-SP1 suppresses mast cell-mediated allergic inflammation *via* inhibition of FcεRI signaling. Front. Immunol., 2020. **11**: p. 50.
